# *Campylobacter jejuni* Is Not Merely a Commensal in Commercial Broiler Chickens and Affects Bird Welfare

**DOI:** 10.1128/mBio.01364-14

**Published:** 2014-07-01

**Authors:** Suzanne Humphrey, Gemma Chaloner, Kirsty Kemmett, Nicola Davidson, Nicola Williams, Anja Kipar, Tom Humphrey, Paul Wigley

**Affiliations:** ^a^ Department of Infection Biology, Institute of Infection and Global Health, University of Liverpool, Leahurst Campus, Neston, Chester, United Kingdom; ^b^ School of Veterinary Science, University of Liverpool, Leahurst Campus, Neston, Chester, United Kingdom; ^c^ Department of Epidemiology and Population Health, Institute of Infection and Global Health, University of Liverpool, Leahurst Campus, Neston, Chester, United Kingdom

## Abstract

*Campylobacter jejuni* is the leading cause of bacterial food-borne infection; chicken meat is its main source. *C. jejuni* is considered commensal in chickens based on experimental models unrepresentative of commercial production. Here we show that the paradigm of *Campylobacter* commensalism in the chicken is flawed. Through experimental infection of four commercial breeds of broiler chickens, we show that breed has a significant effect on *C. jejuni* infection and the immune response of the animals, although these factors have limited impact on the number of bacteria in chicken ceca. All breeds mounted an innate immune response. In some breeds, this response declined when interleukin-10 was expressed, consistent with regulation of the intestinal inflammatory response, and these birds remained healthy. In another breed, there was a prolonged inflammatory response, evidence of damage to gut mucosa, and diarrhea. We show that bird type has a major impact on infection biology of *C. jejuni*. In some breeds, infection leads to disease, and the bacterium cannot be considered a harmless commensal. These findings have implications for the welfare of chickens in commercial production where *C. jejuni* infection is a persistent problem.

## IMPORTANCE

*Campylobacter jejuni* is the most common cause of food-borne bacterial diarrheal disease in the developed world. Chicken is the most common source of infection. *C. jejuni* infection of chickens had previously not been considered to cause disease, and it was thought that *C. jejuni* was part of the normal microbiota of birds. In this work, we show that modern rapidly growing chicken breeds used in intensive production systems have a strong inflammatory response to *C. jejuni* infection that can lead to diarrhea, which, in turn, leads to damage to the feet and legs on the birds due to standing on wet litter. The response and level of disease varied between breeds and is related to regulation of the inflammatory immune response. These findings challenge the paradigm that *C. jejuni* is a harmless commensal of chickens and that *C. jejuni* infection may have substantial impact on animal health and welfare in intensive poultry production**.**

## INTRODUCTION

*Campylobacter jejuni* is the most frequent cause of food-borne bacterial gastroenteritis in the world, estimated to infect 1% of the European Union (EU) population each year. Chicken is the single largest source of infection, with approximately 80% of retail poultry carcasses contaminated in the EU (1). *C. jejuni* colonizes the chicken gut, primarily the large blind ceca at the distal end of the gastrointestinal tract to levels in excess of 10^9^ CFU/g. *Campylobacter* is rapidly transmitted horizontally through broiler (meat-producing) flocks as a consequence of fecal shedding of the bacterium and the coprophagic behavior of the chicken. Control of *C. jejuni* infection focuses on all stages of poultry production and has largely involved attempts to limit exposure of broilers during the rearing cycle and/or carcass treatments to reduce contamination levels at slaughter. To date, no interventions have been shown to be fully successful under commercial conditions. For control to be more effective, it is essential that the infection biology of *C. jejuni* in the chicken is well understood. However, at this time, our understanding is still surprisingly limited. Indeed, the immune response to *C. jejuni* in the chicken and the role that this plays in colonization, pathogenesis, and possible clearance of infection has not been well explored beyond a few descriptive studies.

*C. jejuni* is often considered to be a harmless commensal inhabitant of the chicken gut, and the immune response to it in the intestinal tract is thought to be tolerogenic (2). However, it has been shown previously that *C. jejuni* is recognized by Toll-like receptor 4 (TLR4) and TLR21, the latter being the functional equivalent of mammalian TLR9. This leads to initiation of innate immune responses in the gut that cause an influx of inflammatory cells, including heterophils, the avian equivalent of the neutrophil (3–5). *C. jejuni* infection may also affect the structure of the chicken intestinal epithelium (6). The innate responses lead to adaptive responses that can be measured as both mucosal and systemic specific antibodies (7–9). Studies of T lymphocyte function in the gastrointestinal tract during *C. jejuni* infection are very limited, although T cell responses have been shown in the liver during invasive infections (10). One potential criticism of the past work is that many studies used specific-pathogen-free (SPF) chickens with high challenge doses that have only limited relevance to infection and immunity in commercial broiler breeds. Modern broilers, as exemplified by the A1 and A2 and the C line birds tested in this study, have been bred to grow rapidly and to convert feed into muscle tissue efficiently while keeping high conformity of size and shape to facilitate slaughter and production processes. Such birds reach slaughter weights of approximately 2.2 kg at ~36 days of age. The downside of selective breeding in order to achieve the above production goals are several welfare-associated conditions in these birds, ranging from musculoskeletal disorders to gastrointestinal problems. In contrast, traditional breeds such as the White Leghorn and Light Sussex used in SPF flocks grow far more slowly and have better health but greatly lower productivity levels than modern commercial breeds. Some commercial chicken production systems considered to be “higher welfare” use broilers that grow more slowly, reaching slaughter weight in 50 or more days and usually at reduced stocking densities, typically 30 to 34 kg/m^2^, and with natural light sources. The same breeds may also be used in extensive production. However, such “higher-welfare” systems constitute only a small fraction of total commercial production at present, and faster-growing birds are used in the majority of “standard” intensive production systems where stocking densities of up to 40 kg/m^2^ are employed.

Our previous work on commercial produced broilers found that high levels of hock marks and pododermatitis in flocks were highly significant risk factors for *Campylobacter* colonization. Hock marks or hock burn are marks found on the legs of chickens where ammonia from the waste of other chickens within the litter causes burns. Pododermatitis or foot pad dermatitis is a thickening (keratitis) and discoloration of the foot pad and in more severe cases, lesions of the foot pad of the bird caused through poor-quality or wet litter with high ammonia content. These conditions are more common in the faster-growing broilers, as is *Campylobacter* infection (11, 12). Colles et al. (13) also found an association between hock marks and *Campylobacter* infection in a free-range flock in the United Kingdom. Conditions such as hock marks and pododermatitis can be indicative of poor gut health, leading to wet feces and poor-quality litter that in turn lead to damage of the feet and lower legs of the chickens. It was our assumption that the link with *Campylobacter* was associated with the bacterium better colonizing the damaged gut. This may be the case, but previous preliminary studies have shown that *C. jejuni* directly contributes to poor gut health. These studies found that broilers experimentally infected with *C. jejuni* M1 had significantly higher levels of hock marks and pododermatitis than uninfected controls did. The effects of infection were especially marked in one of the breeds tested (14). We hypothesize that in some modern broiler breeds, *C. jejuni* infection compromises gut health, leading to diarrhea, which indirectly, through wet litter, causes leg health problems in these birds. These conditions are a major bird welfare issue in international chicken production. The aims of the work presented here were to identify mechanisms underlying the observed differences in broiler types in their responses to *C. jejuni* and to examine the impact of infection on gut health and animal welfare.

## RESULTS

### Broiler breed has little impact on *C. jejuni* colonization of the ceca following experimental infection.

Broiler breed had no significant impact on *C. jejuni* M1 levels in ceca following experimental infection of broiler chickens (Table 1), nor was there a statistically significant (*P* = 0.068) impact on *C. jejuni* numbers in this gut compartment at slaughter age (Table 2). Performance data for the birds are summarized in Materials and Methods.

**TABLE 1:** Broiler breed does not significantly affect cecal colonization with *C. jejuni* M1

Breed	Median *C. jejuni* M1 cecal content (CFU ⋅ g−^1^) [range]			Colonization rate (no. of chickens/total no. of chickens)		
	2 dpi	5 dpi	12 dpi	2 dpi	5 dpi	12 dpi
A1	4.62 × 10^7^ [0.00−3.73 × 10^8^]	5.67 × 10^7^ [1.14 × 10^6^−5.00 × 10^8^]	4.34 × 10^7^ [1.74 × 10^6^−1.35 × 10^8^]	9/10	10/10	10/10
A2	3.14 × 10^7^ [1.67 × 10^6^−7.58 × 10^8^]	1.37 × 10^8^ [0.00−3.29 × 10^8^]	5.98 × 10^7^ [5.04 × 10^5^−2.71 × 10^8^]	9/9	7/9	9/9
B1	1.88 × 10^8^ [0.00−7.58 × 10^8^]	1.13 × 10^8^ [0.00−3.04 × 10^9^]	2.20 × 10^7^ [7.44 × 10^5^−1.32 × 10^8^]	8/10	7/10	10/10
B2	1.86 × 10^8^ [2.06 × 10^4^−6.87 × 10^8^]	2.62 × 10^5^ [0.00−7.60 × 10^8^]	1.30 × 10^7^ [0.00−6.36 × 10^7^]	10/10	5/10	9/10

**TABLE 2:** Levels of *C. jejuni* M1 in ceca of broilers grown to commercial slaughter age under experimental conditions

Breed	Slaughter age (days)	Median *C. jejuni* M1 cecal content (CFU ⋅ g^−1^) [range]
A1	35	2.88 × 10^7^ [4.58 × 10^5^−1.93 × 10^8^]
C	36	3.84 × 10^7^ [8.78 × 10^5^−6.86 × 10^8^]
A2	39	4.94 × 10^6^ [3.08 × 10^4^−2.00 × 10^7^]
B1	48	5.30 × 10^6^ [3.38 × 10^3^−2.00 × 10^8^]
B2	56	1.22 × 10^7^ [1.49 × 10^5^−5.77 × 10^8^]

### *C. jejuni* elicits early innate immune activation and an inflammatory response in broilers, which remains elevated for longer in the fast-growing breed A1.

**FIG 1:**
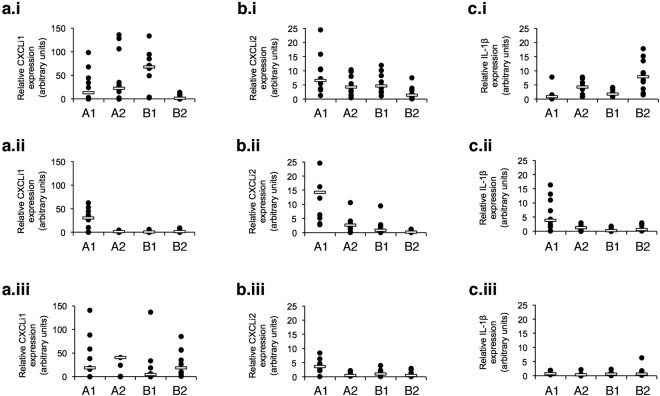
Expression of proinflammatory cytokines and chemokines in cecal tissue following *C. jejuni* M1 infection. (a to c) Fold changes in expression of chemokines CXCLi1 (a) and CXCLi2 (b) and cytokine IL-1β (c) in cecal tissue were examined at 2 (i), 5 (ii), and 12 (iii) dpi. At each time point, group sizes were as follows for the different breeds: *n* = 10 for breed A1, *n* = 9 for breed A2, *n* = 10 for breed B1, and *n* = 10 for breed B2. Each symbol represents the value for an individual chicken. A white bar represents the median value for each group. Significance of differences between the groups was examined using a Kruskal-Wallis test.

**FIG 2:**
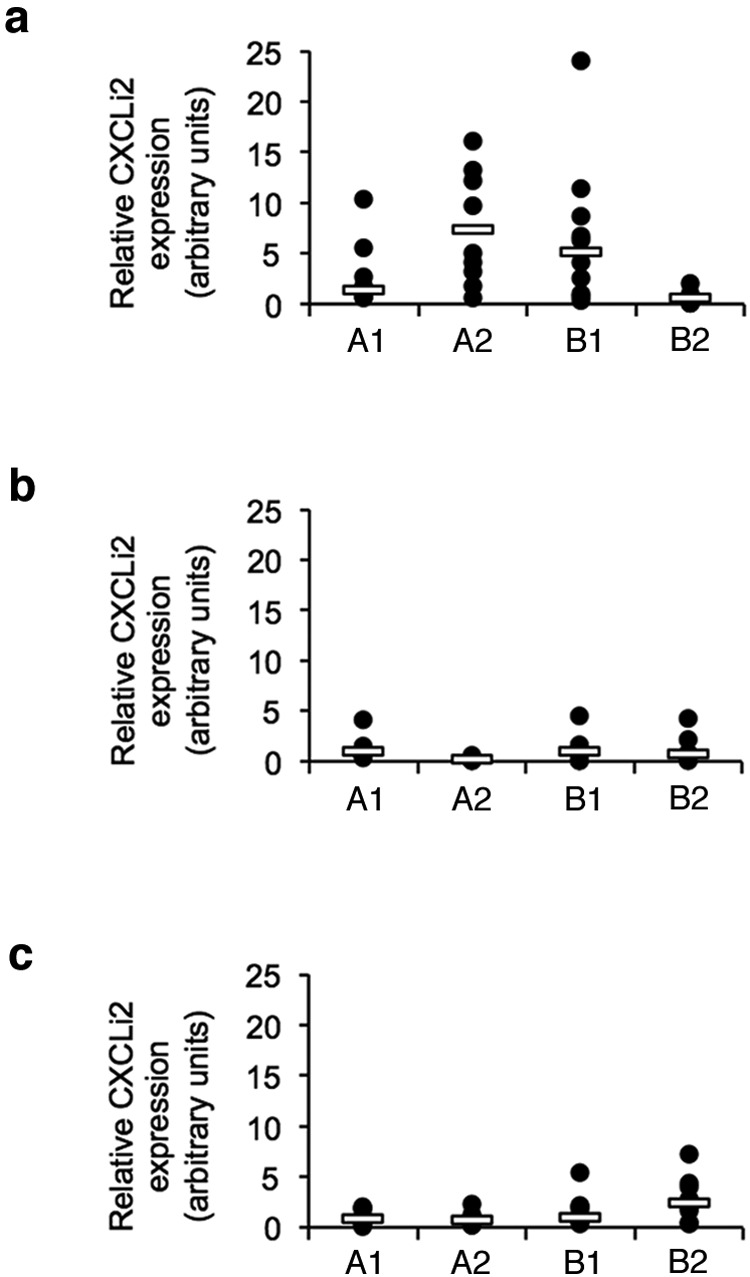
Expression of proinflammatory chemokine CXCLi2 in ileal tissue following *C. jejuni* M1 infection. (a to c) Fold changes in expression of CXCLi2 in ileal tissue were examined at 2 (a), 5 (b), and 12 (c) dpi. At each time point, group sizes were as follows for the different breeds: *n* = 10 for breed A1, *n* = 9 for breed A2, *n* = 10 for breed B1, and *n* = 10 for breed B2. Each symbol represents the value for an individual chicken. A white bar represents the median value for each group. Significance of differences between the groups was examined using a Kruskal-Wallis test.

The innate immune responses to *C. jejuni* M1 was examined in detail in birds from the A1, A2, B1, and B2 lines. Infection elicited expression of the proinflammatory chemokines CXCLi1 (chemokine [C-X-C] motif) ligand i1) (Fig. 1a.i), CXCLi2 (Fig. 1b.i), and interleukin-1β (IL-1β) (Fig. 1c.i) in the ceca of all breeds at 2 days postinfection (dpi). CXCLi2 expression was also detected in the ileum (Fig. 2a). By 5 dpi, these responses were reduced (Fig. 1a.ii,  b.ii, and c.ii) with the exception of the A1 birds in which the levels of expression of all three inflammatory mediators remained significantly higher than in the other birds (CXCLi1 [*P* = 0.017], CXCLi2 [*P* < 0.000], and IL-1β [*P* = 0.012]). By 12 dpi, expression of CXCLi2 remained higher in the *C. jejuni*-infected A1 group than in the other bird types (A2 [*P* = 0.027] and B2 [*P* = 0.019]), although IL-1β expression was reduced (Fig. 1b.iii and c.iii). All four bird breeds showed expression of CXCLi1 at 12 dpi, but with no significant differences (*P* = 0.339) between them (Fig. 1a.iii).

These findings show that the four breeds tested express key inflammatory signals in the innate immune system early after oral infection with *C. jejuni*, but these inflammatory signals are elevated in the A1 birds for much longer. There were no differences in basal constitutive expression of these genes in uninfected control birds of each breed. Considerable variation could be seen in the response between individuals within groups. This is perhaps not surprising, given that these are outbred commercial animals with inherent genetic differences between individuals, although the use of a relatively large group size reduces the impact of such variation.

**FIG 3:**
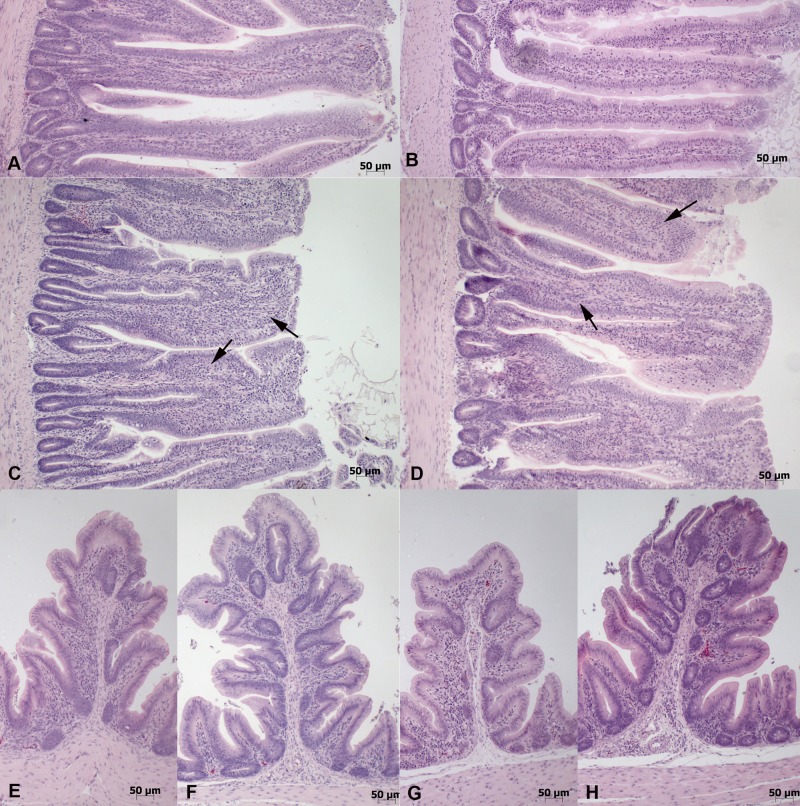
Histological features in control and infected animals at day 5 postinfection. (A to D) Ileum sections from chickens. (A and B) Uninfected control animals of breed A1 (A) and B2 (B). Slim, slender villi with moderate (score 2) lymphocyte and plasma cell infiltration and mild (score 1) heterophil infiltration. (C and D) Infected animals. Villi are thickened (score 2) due to marked (score 3) lymphocyte and plasma cell infiltration and moderate (score 2) heterophil infiltration (arrows) and appear slightly shorter. (E to H) Cecum sections from breed A1 (E and F) and B2 (G and H). Control animals (E and G) exhibit moderate (score 2) lymphocyte and plasma cell infiltration and mild heterophil (score 1) infiltration. In infected animals (F and H), there is increased leukocyte infiltration, with marked (score 3) lymphocyte and plasma cell infiltration and moderate (score 2) heterophil infiltration.

Histological examination of the ilea and ceca of uninfected birds of all breeds identified an apparently constitutive mild diffuse lymphoplasmacellular mucosal infiltration (Fig. 3A, B, E and G). Following infection of the fastest (A1) and slowest (B2) growing birds, increased numbers of inflammatory cells, heterophils, and lymphocytes were seen (Fig. 3C, D, F, and H), commensurate with the cytokine responses seen at 5 dpi. In both breeds, there was evidence of mucosal damage, notably thickening, shortening, and fusion of villi in the ileum (Fig. 3C and D). This was more frequently observed and more intense in the A1 breed where such pathological changes were seen in 7 of 10 birds. In contrast, in the B2 group, only 3 of 10 birds showed morphological evidence of mucosal damage and inflammation. A similar pattern was seen at 12 dpi. These data indicate that the infection resulted in damage and inflammatory infiltration of the intestinal mucosa of both bird types, but that they were more common and more intense in the faster-growing breed (A1).

### Prolonged inflammation in the intestines of faster-growing breeds of chickens results in diarrhea and affects their welfare.

By 12 dpi, the infected A1 birds were found to have diarrhea when examined during twice-daily welfare checks and at ante mortem examination where fecal pasting of feathers around the cloaca consistent with diarrhea was found. Similar observations were also made in C birds infected to examine longer-term carriage. Although feces were generally wetter in A1 birds than in other breeds, no diarrhea was seen in the control group. Diarrhea was not seen in the A2 or B birds. At postmortem examination, A1 birds were found to have more watery intestinal contents and some evidence of inflammation, such as mild hyperemia of the cecal wall. Diarrhea in the A1 birds corresponds to the greater and prolonged inflammatory response seen in this breed. Furthermore, despite birds being maintained under experimental conditions, including regular changes of litter, 9 of 10 *C. jejuni*-infected A1 birds showed signs of pododermatitis at 12 dpi. In contrast, only 2 of 10 infected B1 bird and 2 of 9 infected A2 birds had pododermatitis. This condition was not seen in the infected B2 group.

### Disease in some broiler breeds is a consequence of immune dysregulation in the intestine.

**FIG 4:**
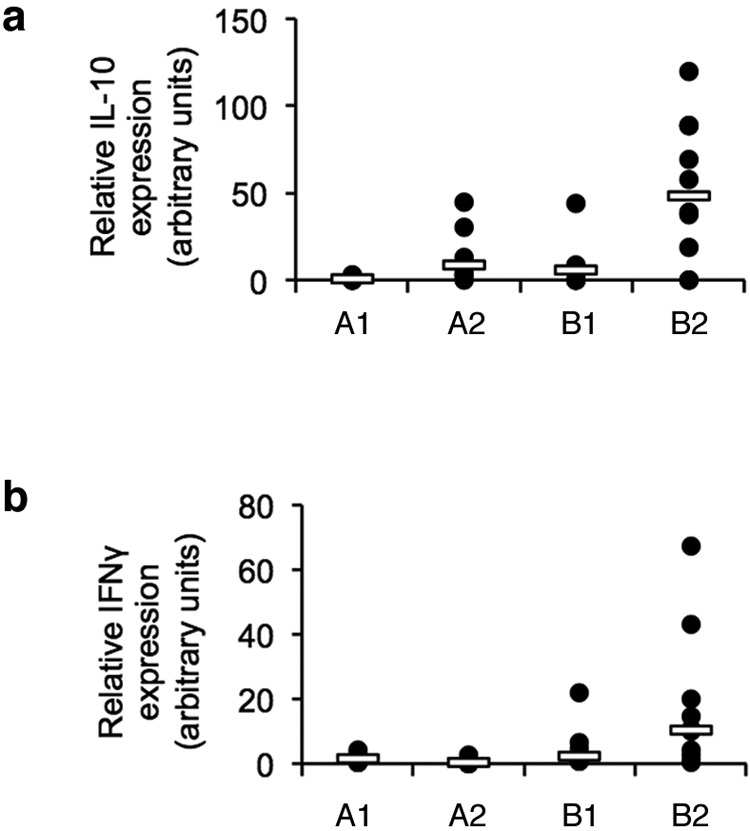
Expression of T_H_1 and regulatory cytokines in cecal tissue following *C. jejuni* M1 infection. (a) Fold changes in expression of the regulatory cytokine IL-10 in tissue were examined at 12 dpi. (b) Fold changes in expression of the T_H_1-biased cytokine IFN-γ were also examined at 12 dpi. At each time point, group sizes were as follows for the different breeds: *n* = 10 for breed A1, *n* = 9 for breed A2, *n* = 10 for breed B1, and *n* = 10 for breed B2. Each symbol represents the value for an individual chicken. A white bar represents the median value for each group. Significance of differences between the groups was examined using a Kruskal-Wallis test.

Differential expression of IL-10 in breeds was seen in the ceca at 12 dpi (Fig. 4a) with a median 50-fold increase in expression in the slower-growing B2 birds, significantly higher than the minimal levels in the A1 animals (*P* = 0.003) (see Fig. S1 in the supplemental material). Expression in the A2 and B1 birds was intermediate between these extremes. IL-10 is a key regulatory cytokine in controlling inflammation. The absence of IL-10 expression in the A1 birds indicates that inflammatory responses in the intestine are poorly regulated, leading to prolonged inflammation and diarrhea, as described above. A similar, but not statistically significant (*P* = 0.068) pattern of expression was also seen for gamma interferon (IFN-γ) (Fig. 4B), suggesting that there may be further variation between breeds in their T lymphocyte function (see Fig. S2 and S3 in the supplemental material).

Although there was variation in inflammatory and regulatory responses in the gut, all breeds produced specific IgM and IgG antibodies at similar titers following infection (data not shown).

## DISCUSSION

Our work shows that *C. jejuni* cannot be regarded as simply a harmless gut commensal of some modern commercial broiler chickens. Infection of broiler chickens is clearly associated with intestinal inflammation. While the possibility of this response involving some interaction with other bacteria in the intestinal microbiota cannot be ruled out, it is clear that the infection with *C. jejuni* is essential in causing the inflammation described. In the fast-growing A1 breed, this inflammation is prolonged, leading to diarrhea and subsequent associated pathology to the feet and legs of these birds. Diarrhea was also seen in breed C birds, but detailed innate immune responses in this breed were not examined because of logistical constraints. Detailed examination of responses to infection in four broiler breeds found that *C. jejuni* M1 elicited an inflammatory innate immune response in the intestinal tract, which led to a specific antibody response. Intestinal disease in the A1 birds appears to be a consequence of a poorly regulated immune response, as expression levels of proinflammatory signals such as CXCL chemokines and IL-1β remain higher than in the three other breeds at 5 and 12 dpi. In contrast, IL-10 expression is significantly higher in B2 birds at 12 dpi, suggesting that it is much better able to regulate its inflammatory response and that dysregulation of this in A1 birds leads to infection-mediated pathology in the lower intestinal tract. The importance of IL-10 in regulating *C. jejuni* enteritis has been shown in the C57BL/6 IL-10^−/−^ mice used as model for acute enterocolitis (15, 16). While both control and IL-10-deficient C57BL/6 mice can be colonized by *C. jejuni*, only C57BL/6 IL-10^−/−^ mice develop enteritis (17).

The data presented here show that there is no intrinsic difference in the susceptibility of broiler breeds to *C. jejuni* under experimental conditions. As such, it is unclear as to whether any change in the current broiler breed type will have any impact on public health. However, breeding chickens resistant to *Campylobacter* infection is considered, along with vaccination, to be a potential long-term intervention in controlling these bacteria in chicken production (18, 19). Studies using inbred and outbred lines of chicken have indicated that differences in innate immunity, particularly β-defensins secreted in the gut, may hold the key to resistance (20). However, more-detailed transcriptional studies of the response to *C. jejuni* in two broiler lines differing in resistance to colonization indicate that there is greater activity of regulatory T lymphocytes in the more resistant line (20, 21). T regulatory cells (or TRegs) are important in maintaining gut homeostasis and regulating the response to infection of the chicken and other species through production of cytokines such as IL-10 and tumor growth factor beta (TGF-β). Taken with our data on expression of IL-10, it appears that regulation of responses through T regulatory lymphocytes is key to both maintaining a healthy gut and reducing colonization by *C. jejuni* following infection. As such, we suggest that certain faster-growing breeds of broiler chicken, such as the A1 breed, have a functional innate response to *Campylobacter* infection but do not have an effective T regulatory one, leading to the pathological changes we have seen. This dysfunction, if it occurs with other enteric pathogens, may also lead to inflammatory damage in the intestinal tract under production conditions. In our past work (11, 12), we did not ascertain whether pododermatitis and hock marks, linked to an increased risk of *Campylobacter* infection, preceded infection or occurred because of it. Data presented here show that *C. jejuni* makes a direct contribution to this condition. The immune dysfunction we have seen in response to *C. jejuni* does not seem to be linked to more rapid growth *per se* and is more likely to be related to the genetic background of the birds, as this will differ even in animals bred by the same company. Such information is commercially sensitive and therefore not freely available.

In summary, we show that *C. jejuni* is not merely a harmless commensal of the broiler chicken and that infection can lead to diarrhea and inflammatory damage in the birds. Although previous studies have shown that there is an inflammatory response to *Campylobacter* infection in the chicken, the immune response in the gut has been considered largely “tolerogenic,” allowing the bacteria to remain in the intestine in the absence of any pathological changes or systemic infection. Indeed, it may be that the “natural” immune response to *C. jejuni* in the chicken is centered upon confining the pathogen to the intestine where it persists and that the response in the gut is tightly regulated to prevent excessive inflammation, which in traditional chicken breeds leads to little or no ill effect on the bird. However, in modern broiler breeds, the drive toward improved productivity and carcass conformity would appear to have had a negative impact on T lymphocyte function, leading to a loss of immune regulation in the gut. The result of this imbalance is that *C. jejuni* infection results in poorly controlled inflammation, leading to intestinal mucosal damage and diarrhea.

## MATERIALS AND METHODS

### Bacterial strains and culture conditions.

*C. jejuni* M1 was kindly provided by Lisa Williams (University of Bristol). Bacteria were grown from stocks maintained at −80°C on Columbia blood agar (Lab M, Heywood, Lancashire, United Kingdom) supplemented with 5% defibrinated horse blood (Oxoid, Basingstoke, Hampshire, United Kingdom) for 48 h in microaerobic conditions (80% N_2_, 12% CO_2_, 5% O_2_, and 3% H_2_) at 41.5°C. Liquid cultures were grown for 24 h in 10 ml of Mueller-Hinton broth (MHB) in microaerobic conditions at 41.5°C and adjusted by dilution in fresh MHB to a final concentration of 10^6^ CFU/ml.

### Experimental animals.

All work was conducted in accordance with United Kingdom legislation governing experimental animals under project licenses PPL 40/3063 (experiment 1) and 40/3652 (experiment 2) and was approved by the University of Liverpool ethical review process prior to the award of the licenses. All animals were checked a minimum of twice daily to ensure their health and welfare. Five common broiler breeds were used. Data from the breeding companies state that the A1 bird reaches live slaughter weight (2.2 kg) at 36 days of age, as does the breed C bird. The A2 bird reaches 2.2 kg at 37 days, while the B1 and B2 birds take 48 and 56 days to reach this weight, respectively. Not all breeds were used in all experiments, and the decision to use breed A birds for detailed examination, rather than the C line, was based on current market share in the United Kingdom, where many more breed A birds are reared.

Age-matched, 1-day-old chicks of broiler chickens were obtained from a commercial hatchery. Chicks were housed in the University of Liverpool high-biosecurity poultry unit. Chicks were maintained in floor pens at stocking levels recommended by the Home Office, allowing floor space of 2,000 cm^2^ per bird for all groups and were given *ad libitum* access to water and a pelleted laboratory-grade vegetable protein-based diet (SDS, Witham, Essex, United Kingdom) with a feeder and drinker available per 15 birds. Chicks were housed in separate groups at a temperature of 30°C, which was reduced to 20°C at 3 weeks of age. Each infected group was housed in a single room with filtered air supply with lobbied entry to each room and dedicated protective clothing and boots. Two control groups were housed in separate pens in an individual room. Prior to experimental infection, all birds were confirmed as *Campylobacter* free by taking cloacal swabs, which were streaked onto selective blood-free agar (modified charcoal-cefoperazone-deoxycholate agar [mCCDA]) supplemented with *Campylobacter* Enrichment Supplement (SV59; Mast Group, Bootle, Merseyside, United Kingdom) and grown for 48 h in microaerobic conditions at 41.5°C. All microbiological media were purchased from Lab M (Heywood, Lancashire, United Kingdom). Throughout the experiment, litter was changed every 4 days, and pens were cleaned to limit retransmission within groups and to reduce problems associated with wet litter.

### Effect of broiler breed on *C. jejuni* infection (experiment 1).

When the chicks were 21 days old, A1 (*n* = 40), A2 (*n* = 38), B1 (*n* = 40), and B2 (*n* = 40) broilers were orally infected with 10^5^ cells of *C. jejuni* M1 in 0.2 ml of MHB. Control birds (A1 [*n* = 16], A2 [*n* = 15], B1 [*n* = 15], and B2 [*n* = 16]) received 0.2 ml of sterile MHB. At 2, 5, and 12 dpi, 10 infected birds and 5 control birds of each breed were killed by cervical dislocation. At postmortem examination, samples of tissue and gut contents were taken and processed for host gene expression analysis, histology, and *Campylobacter* enumeration.

### Assessment of *C. jejuni* load.

In order to determine the levels of *C. jejuni* colonization in each of the breeds, cecal contents were collected from individual birds at necropsy and diluted in 9 volumes of maximal recovery diluent (MRD). Serial 10-fold dilutions were made of each sample in MRD, and using the method of Miles et al. (22), duplicate 50-µl spots were plated onto mCCDA agar supplemented with SV59. The plates were incubated under microaerobic conditions at 41.5°C for 48 h, and *Campylobacter* colonies were enumerated to give CFU/g of cecal contents.

### RNA extraction and real-time qRT-PCR.

Samples of ileal and cecal tissue from infected and control birds were collected and stored in 500 µl of RNAlater at −20°C (Sigma, Poole, Dorset, United Kingdom). Total RNA was isolated from 20 to 30 mg of tissue using an RNeasy minikit (Qiagen, West Sussex, United Kingdom) according to the manufacturer’s instructions. Isolated RNA was eluted into 50 µl RNase-free water and stored at −80°C. The yield of total RNA was determined using a NanoDrop (ND-1000) spectrophotometer.

Expression of mRNA for the cytokines interleukin-1β (IL-1β), gamma interferon (IFN-γ), IL-10, and IL-4 and the chemokines CXCLi1 (chemokine [C-X-C motif] ligand i1) and CXCLi2 in these tissues was measured by real-time quantitative reverse transcription-PCR (qRT-PCR), using primer and probe sequences described by Shini and Kaiser (23). 28S rRNA was used as the housekeeping gene. Twenty-microliter  reaction mixtures for one-step qRT-PCR were prepared using the RotorGene Probe RT-PCR kit (Qiagen, West Sussex, United Kingdom) and contained 1 µl of total RNA (at 20 ng/µl), 10 µl of RotorGene Probe RT-PCR master mix, 0.2 µl of RotorGene reverse transcriptase enzyme mix, 1.6 µl of each primer (at 10 µM), 0.8 µl of probe (at 5 µM), and 4.8 µl of RNase-free water. Triplicate reactions were set up for each sample using an automated QIAgility system (Qiagen, West Sussex, United Kingdom). Real-time RT-PCR was performed on a RotorGene Q system (Qiagen, West Sussex, United Kingdom) with the following reaction profile: 50°C for 10 min to facilitate reverse transcription of RNA to cDNA, followed by 95°C for 5 min, and 40 repeat cycles, with 1 cycle consisting of 95°C for 5 s and 60°C for 10 s.

Expression of the target gene was determined using the cycle threshold (*C*_*T*_) value relative to that for the 28S rRNA reference gene (Δ*C*_*T*_). Results are expressed as fold changes in corrected target gene expression (Δ*C*_*T*_) in infected animals relative to the control animals (2^−ΔΔCT^).

### Histology.

Samples of ileum and cecum from the two breeds displaying the most extreme differences in cytokine response in the experiments, A1 and B2, were examined for histological assessment. These samples were obtained from the same infected and uninfected birds used for cytokine analysis. Tissue samples were fixed in 4% (wt/vol) paraformaldehyde in phosphate-buffered saline (PBS) and routinely embedded in paraffin wax. Sections (3 to 5 µm thick) were prepared and stained with hematoxylin and eosin (H&E) using standard protocols. Tissue sections were assessed in a blind manner by veterinary pathologists for any pathological changes and the semiquantitative analysis of the inflammatory infiltrate, represented by grading the degree of heterophil and lymphoplasmacellular infiltration as follows: 1 for slight infiltration, 2 for mild infiltration, 3 for moderate infiltration, 4 for marked infiltration, and 5 for severe infiltration.

### *C. jejuni* carriage at slaughter age (experiment 2).

When the chicks were 21 days old, A1 (*n* = 7), C (*n* = 9), A2 (*n* = 9), B1 (*n* = 9), and B2 (*n* = 10) birds were orally infected with 10^5^ cells of *C. jejuni* M1 in 0.2 ml of MHB. The birds were grown to their commercial slaughter ages (36 days for A1 breed, 36 days for C, 37 days for A2, 48 days for B1, and 56 days for B2]) and were killed by cervical dislocation. At postmortem examination, the contents were taken from both ceca for *Campylobacter* enumeration using the method described above. Original groups contained 10 birds, but because of welfare concerns unrelated to *C. jejuni* infection, some birds were culled prior to the end of the experiment.

### Statistical analyses.

Statistical analyses were performed using SPSS v20 (IBM). For comparison of bacterial loads between infected groups and fold changes in cytokine expression between breeds, the Kruskal-Wallis (nonparametric analysis of variance [ANOVA]) test was applied, as the data were not normally distributed. Differences were considered significant where *P* < 0.05.

## SUPPLEMENTAL MATERIAL

Figure S1 Expression of IL-10 in ileal tissue following *C. jejuni* M1 infection. (a to c) Fold changes in expression of IL-10 in ileal tissue were examined at 2 (a), 5 (b) and 12 (c) dpi. At each time point, group sizes were as follows for the different breeds: *n* = 10 for breed A1, *n* = 9 for breed A2, *n* = 10 for breed B1, and *n* = 10 for breed B2. Each symbol represents the value for an individual chicken. Each bar represents the median value for each group. Significance of differences between the groups was examined using a Kruskal-Wallis test. Download Figure S1, TIF file, 4.4 MB

Figure S2 Expression of IFN-γ in ileal tissue following *C. jejuni* M1 infection. (a to c) Fold changes in expression of IFN-γ in ileal tissue were examined at 2 (a), 5 (b) and 12 (c) dpi. At each time point, group sizes were as follows for the different breeds: *n* = 10 for breed A1, *n* = 9 for breed A2, *n* = 10 for breed B1, and *n* = 10 for breed B2. Each symbol represents the value for an individual chicken. Each bar represents the median value for each group. Significance of differences between the groups was examined using a Kruskal-Wallis test. Download Figure S2, TIF file, 4.4 MB

Figure S3 Expression of IL-4 in cecal tissue following *C. jejuni* M1 infection. (a to c) Fold changes in expression of IL-4 in cecal tissue were examined at 2 (a), 5 (b), and 12 (c) dpi. At each time point, group sizes were as follows for the different breeds: *n* = 10 for breed A1, *n* = 9 for breed A2, *n* = 10 for breed B1, and *n* = 10 for breed B2. Each symbol represents the value for an individual chicken. Each bar represents the median value for each group.Significance of differences between the groups was examined using a Kruskal-Wallis test. Download Figure S3, TIF file, 4.4 MB

## References

[1] European Food Safety Authority 2010 Analysis of the baseline survey on the prevalence of *Campylobacter* in broiler batches and of *Campylobacter* and *Salmonella* on broiler carcasses in the EU, 2008 - Part A: *Campylobacter* and *Salmonella* prevalence estimates. EFSA J. Avgust:1503–1602. 10.2903/j.efsa.2010.1503

[2] Hermans D Pasmans F Heyndrickx M Van Immerseel F Martel A Van Deun K Haesebrouck F 2012 A tolerogenic mucosal immune response leads to persistent Campylobacter jejuni colonization in the chicken gut. Crit. Rev. Microbiol. 38:17–29. 10.3109/1040841X.2011.615298 21995731

[3] de Zoete MR Keestra AM Roszczenko P van Putten JP 2010 Activation of human and chicken Toll-like receptors by *Campylobacter* spp. Infect. Immun. 78:1229–1238. 10.1128/IAI.00897-09 20038539 PMC2825908

[4] Meade KG Narciandi F Cahalane S Reiman C Allan B O’Farrelly C 2009 Comparative in vivo infection models yield insights on early host immune response to Campylobacter in chickens. Immunogenetics 61:101–110. 10.1007/s00251-008-0346-7 19082824

[5] Smith CK Abuoun M Cawthraw SA Humphrey TJ Rothwell L Kaiser P Barrow PA Jones MA 2008 Campylobacter colonization of the chicken induces a proinflammatory response in mucosal tissues. FEMS Immunol. Med. Microbiol. 54:114–121. 10.1111/j.1574-695X.2008.00458.x 18647351

[6] Awad WA Molnar A Aschenbach JR Ghareeb K Khayal B Hess C Liebhart D Dublecz K Hess M 19 February 2014 *Campylobacter* infection in chickens modulates the intestinal epithelial barrier function. Innate Immun. 10.1177/1753425914521648 24553586

[7] Widders PR Thomas LM Long KA Tokhi MA Panaccio M Apos E 1998 The specificity of antibody in chickens immunised to reduce intestinal colonisation with Campylobacter jejuni. Vet. Microbiol. 64:39–50. 10.1016/S0378-1135(98)00251-X 9874102

[8] Cawthraw S Ayling R Nuijten P Wassenaar T Newell DG 1994 Isotype, specificity, and kinetics of systemic and mucosal antibodies to Campylobacter jejuni antigens, including flagellin, during experimental oral infections of chickens. Avian Dis. 38:341–349. 10.2307/1591960 7526839

[9] de Zoete MR van Putten JP Wagenaar JA 2007 Vaccination of chickens against Campylobacter. Vaccine 25:5548–5557. 10.1016/j.vaccine.2006.12.002 17224215

[10] Jennings JL Sait LC Perrett CA Foster C Williams LK Humphrey TJ Cogan TA 2011 Campylobacter jejuni is associated with, but not sufficient to cause vibrionic hepatitis in chickens. Vet. Microbiol. 149:193–199. 10.1016/j.vetmic.2010.11.005 21112163

[11] Rushton SP Humphrey TJ Shirley MDF Bull S Jørgensen F 2009 Campylobacter in housed broiler chickens: a longitudinal study of risk factors. Epidemiol. Infect. 137:1099–1110. 10.1017/S095026880800188X 19149909

[12] Bull SA Thomas A Humphrey T Ellis-Iversen J Cook AJ Lovell R Jorgensen F 2008 Flock health indicators and *Campylobacter* spp. in commercial housed broilers reared in Great Britain. Appl. Environ. Microbiol. 74:5408–5413. 10.1128/AEM.00462-08 18641155 PMC2546635

[13] Colles FM Jones TA McCarthy ND Sheppard SK Cody AJ Dingle KE Dawkins MS Maiden MC 2008 Campylobacter infection of chickens in a free-range environment. Environ. Microbiol. 10:2042–2050 18412548 10.1111/j.1462-2920.2008.01623.x PMC2702501

[14] Williams LK Sait LC Trantham EK Cogan TA Humphrey TJ 2013 Campylobacter infection has different outcomes in fast- and slow-growing broiler chickens. Avian Dis. 57:238–241. 10.1637/10442-110212-Reg.1 24689180

[15] Mansfield LS Bell JA Wilson DL Murphy AJ Elsheikha HM Rathinam VA Fierro BR Linz JE Young VB 2007 C57BL/6 and congenic interleukin-10-deficient mice can serve as models of *Campylobacter jejuni* colonization and enteritis. Infect. Immun. 75:1099–1115. 10.1128/IAI.00833-06 17130251 PMC1828563

[16] Haag LM Fischer A Otto B Plickert R Kühl AA Göbel UB Bereswill S Heimesaat MM 2012 Campylobacter jejuni induces acute enterocolitis in gnotobiotic IL-10-/- mice via Toll-like-receptor-2 and -4 signaling. PLoS One 7:e40761. 10.1371/journal.pone.0040761 22808254 PMC3393706

[17] Mansfield LS Patterson JS Fierro BR Murphy AJ Rathinam VA Kopper JJ Barbu NI Onifade TJ Bell JA 2008 Genetic background of IL-10^−/−^ mice alters host-pathogen interactions with Campylobacter jejuni and influences disease phenotype. Microb. Pathog. 45:241–257. 10.1016/j.micpath.2008.05.010 18586081 PMC4148907

[18] Calenge F Beaumont C 2012 Toward integrative genomics study of genetic resistance to Salmonella and Campylobacter intestinal colonization in fowl. Front. Genet. 3:261. 10.3389/fgene.2012.00261 23412643 PMC3571208

[19] Kaiser P Howell MM Fife M Sadeyen JR Salmon N Rothwell L Young J Poh TY Stevens M Smith J Burt D Swaggerty C Kogut M 2009 Towards the selection of chickens resistant to Salmonella and Campylobacter infections. Bull. Mem. Acad. R. Med. Belg. 164:17–26 19718951

[20] Li X Swaggerty CL Kogut MH Chiang HI Wang Y Genovese KJ He H Zhou H 2010 Gene expression profiling of the local cecal response of genetic chicken lines that differ in their susceptibility to Campylobacter jejuni colonization. PLoS One 5:e11827. 10.1371/journal.pone.0011827 20676366 PMC2911375

[21] Li XY Swaggerty CL Kogut MH Chiang HI Wang Y Genovese KJ He H Pevzner IY Zhou HJ 2011 Caecal transcriptome analysis of colonized and non-colonized chickens within two genetic lines that differ in caecal colonization by Campylobacter jejuni. Anim. Genet. 42:491–500. 10.1111/j.1365-2052.2010.02168.x 21906100

[22] Miles AA Misra SS Irwin JO 1938 The estimation of the bactericidal power of the blood. J. Hyg. (Lond) 38: 732–749 20475467 10.1017/s002217240001158x PMC2199673

[23] Shini S Kaiser P 2009 Effects of stress, mimicked by administration of corticosterone in drinking water, on the expression of chicken cytokine and chemokine genes in lymphocytes. Stress 12:388–399. 10.1080/10253890802526894 19006006

