# Supplementary figures and images for: Campylobacter jejuni Is Not Merely a Commensal in Commercial Broiler Chickens and Affects Bird Welfare
Source: mBio. 2014 Jul 1;5(4):e01364-14. doi: 10.1128/mBio.01364-14 (PMC4161246; doi:10.1128/mBio.01364-14)

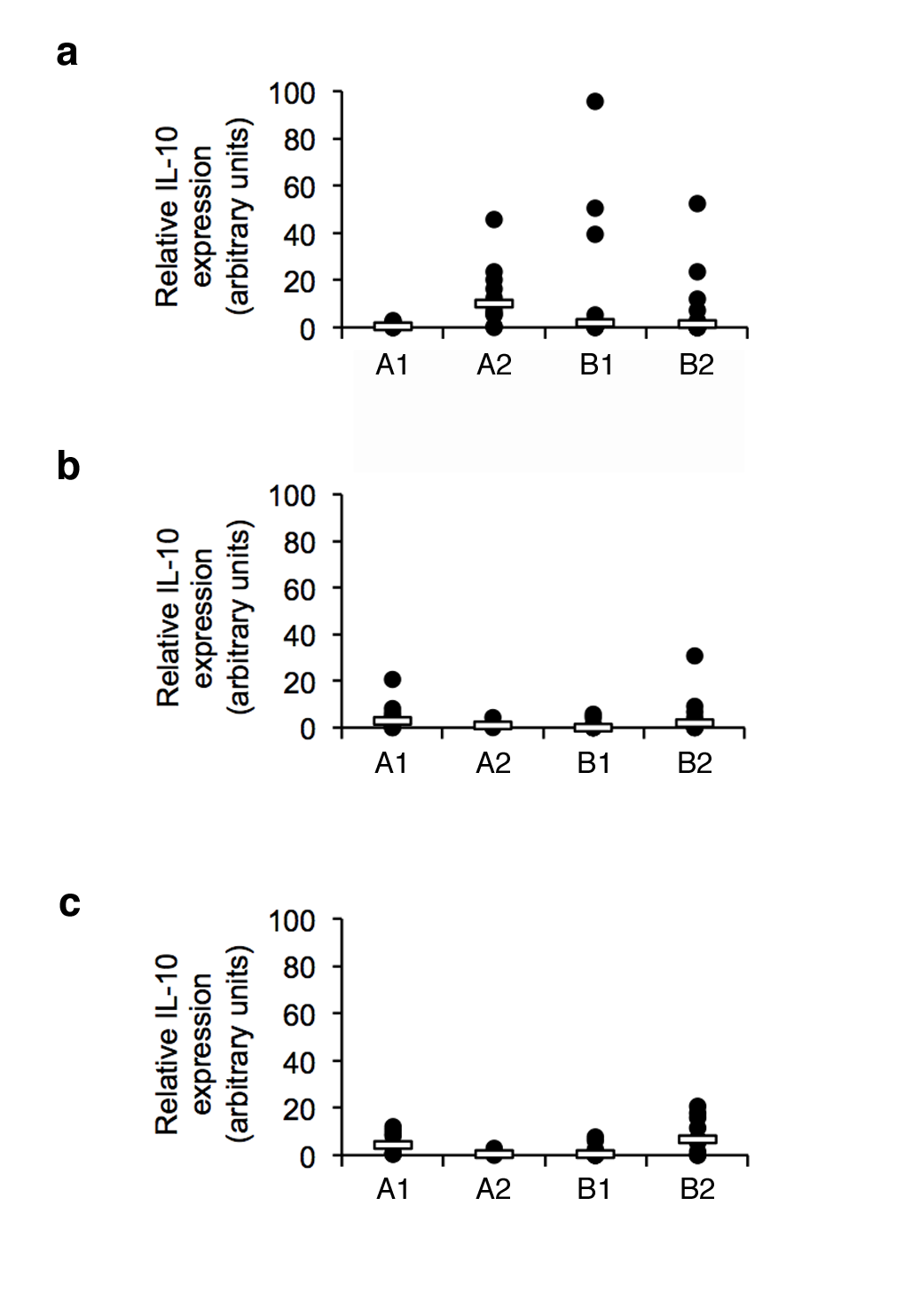

Supplement: Figure S1 — Expression of IL-10 in ileal tissue following C. jejuni M1 infection. (a to c) Fold changes in expression of IL-10 in ileal tissue were examined at 2 (a), 5 (b) and 12 (c) dpi. At each time point, group sizes were as follows for the different breeds: n = 10 for breed A1, n = 9 for breed A2, n = 10 for breed B1, and n = 10 for breed B2. Each symbol represents the value for an individual chicken. Each bar represents the median value for each group. Significance of differences between the groups was examined using a Kruskal-Wallis test. Download [file mbo003141883sf01.tif]

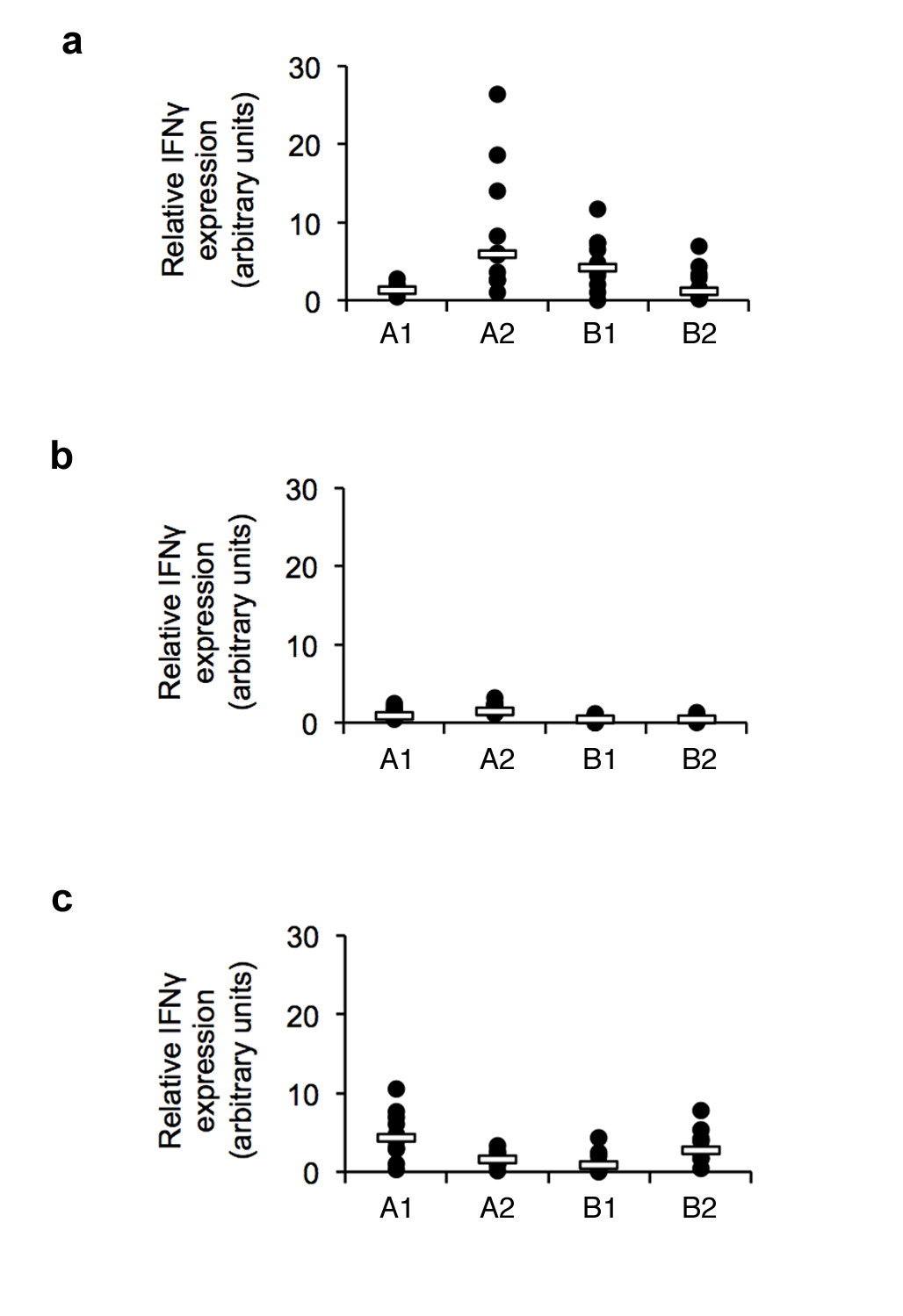

Supplement: Figure S2 — Expression of IFN-γ in ileal tissue following C. jejuni M1 infection. (a to c) Fold changes in expression of IFN-γ in ileal tissue were examined at 2 (a), 5 (b) and 12 (c) dpi. At each time point, group sizes were as follows for the different breeds: n = 10 for breed A1, n = 9 for breed A2, n = 10 for breed B1, and n = 10 for breed B2. Each symbol represents the value for an individual chicken. Each bar represents the median value for each group. Significance of differences between the groups was examined using a Kruskal-Wallis test. Download [file mbo003141883sf02.tif]

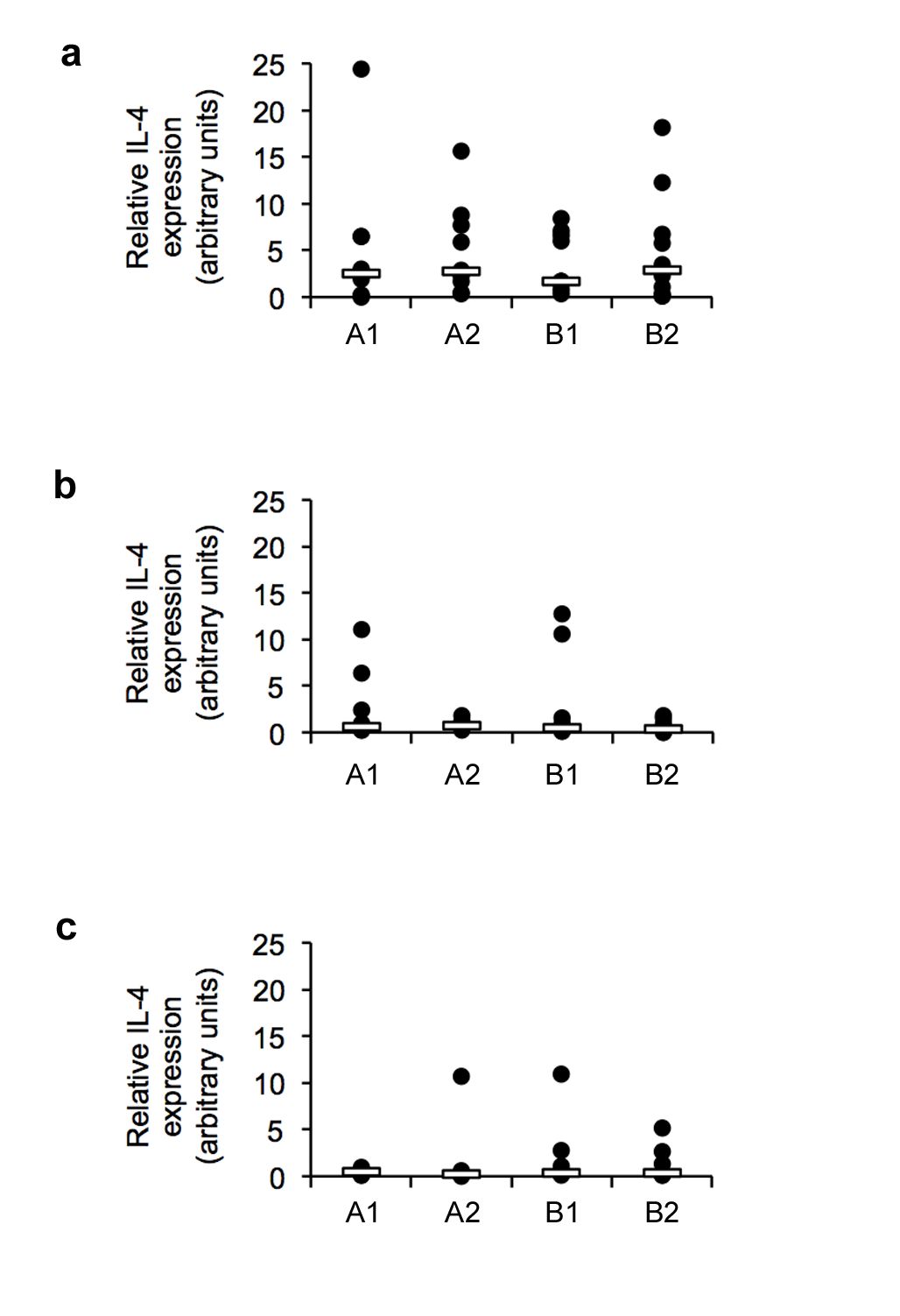

Supplement: Figure S3 — Expression of IL-4 in cecal tissue following C. jejuni M1 infection. (a to c) Fold changes in expression of IL-4 in cecal tissue were examined at 2 (a), 5 (b), and 12 (c) dpi. At each time point, group sizes were as follows for the different breeds: n = 10 for breed A1, n = 9 for breed A2, n = 10 for breed B1, and n = 10 for breed B2. Each symbol represents the value for an individual chicken. Each bar represents the median value for each group.Significance of differences between the groups was examined using a Kruskal-Wallis test. Download [file mbo003141883sf03.tif]
